# From Zero to Hero: The Cyanide-Free Formation of Amino Acids and Amides from Acetylene, Ammonia and Carbon Monoxide in Aqueous Environments in a Simulated Hadean Scenario

**DOI:** 10.3390/life14060719

**Published:** 2024-06-01

**Authors:** Christian Seitz, Thomas Geisberger, Alexander Richard West, Jessica Fertl, Wolfgang Eisenreich, Claudia Huber

**Affiliations:** Bayerisches NMR Zentrum, Strukturelle Membranbiochemie, Technische Universität München, Lichtenbergstr. 4, 85748 Garching, Germany; c.seitz@tum.de (C.S.); thomas.geisberger@t-online.de (T.G.); a.r.west@student.rug.nl (A.R.W.); jessi-fertl@web.de (J.F.); wolfgang.eisenreich@mytum.de (W.E.)

**Keywords:** amino acids, amide, peptide bond, acetylene, transition metal sulfides, hydrothermal conditions, origin of life

## Abstract

Amino acids are one of the most important building blocks of life. During the biochemical process of translation, cells sequentially connect amino acids via amide bonds to synthesize proteins, using the genetic information in messenger RNA (mRNA) as a template. From a prebiotic perspective (i.e., without enzymatic catalysis), joining amino acids to peptides via amide bonds is difficult due to the highly endergonic nature of the condensation reaction. We show here that amides can be formed in reactions catalyzed by the transition metal sulfides from acetylene, carbon monoxide and ammonia under aqueous conditions. Some α- and β-amino acids were also formed under the same conditions, demonstrating an alternative cyanide-free path for the formation of amino acids in prebiotic environments. Experiments performed with stable isotope labeled precursors, like ^15^NH_4_Cl and ^13^C-acetylene, enabled the accurate mass spectroscopic identification of the products formed from the starting materials and their composition. Reactions catalyzed using the transition metal sulfides seem to offer a promising alternative pathway for the formation of amides and amino acids in prebiotic environments, bypassing the challenges posed by the highly endergonic condensation reaction. These findings shed light on the potential mechanisms by which the building blocks of life could have originated on early Earth.

## 1. Introduction

The role of amino acids as the fundamental building blocks of life is paramount in both modern biochemistry and theories of the origin of life. For modern biochemistry, α-amino acids are essential building blocks for peptides and proteins. In the context of the origin of life, various abiotic conditions, whether terrestrial or extraterrestrial in nature, have been proposed to explain their formation. One of the first experiments, which synthesized organic compounds such as amino acids from an inorganic starting material, were the experiments by Miller and Urey [1,2]. These pioneers of research on the origin of life used electric discharges as an energy form in a reducing gas atmosphere consisting of NH_3_, H_2_ and CH_4_. Additionally, later, there were also experiments using a more neutral atmosphere consisting of CO_2_, CO, N_2_ and H_2_O; however, the yields were not as high as the ones in a reducing gas atmosphere [3].

In the meantime, many other types of energy sources were considered to form organic compounds from simple precursors on the early Earth. There have been attempts using UV [4,5,6,7,8], X-ray [9,10] and proton irradiation [11,12], shock heating from 90 °C [13] to over 200 °C [14,15,16] or using volcanism-induced electric discharges as other possible energy sources [17,18]. In the iron–sulfur theory of the origin of metabolism [19], chemical energy acts in the formation of amino acids [20] and peptides [21] using FeS/NiS catalysts. Herrera, another pioneer in the origin of life field, simply mixed ammonium thiocyanate with formaline and discovered amino acids and further organic molecules [22].

However, amino acids could also have originated from extra-terrestrial sources. Amino acids were detected in carbonaceous meteorites of various types from C1 [23,24,25], through to C2 [26,27,28] and C3 [24,29,30]. Recently, glycine oligopeptides were synthesized from C, CO and NH_3_ under simulated stellar conditions [31].

**Scheme 1:**
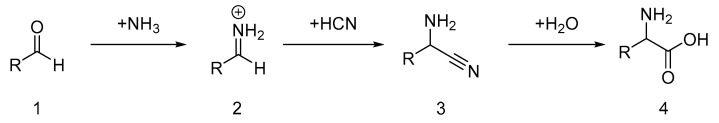
Basic process of the Strecker reaction. The reaction proceeds via the nucleophilic addition of ammonia to the aldehyde (1). This generally extends to the iminium ion (2). The cyanide adds to this electrophilic species, resulting in an α-aminonitrile (3). The amino acid (4) is ultimately formed through hydrolysis.

The prebiotic synthesis of amino acids likely follows a Strecker reaction [32], starting from an aldehyde (Scheme 1). With the addition of ammonia, an imine is built, which reacts with cyanide to form α-aminonitrile which is finally hydrolyzed to an α-amino acid. Depending on the structure of R in the starting aldehyde, different amino acids can be synthesized by this type of reaction [33].

In nature, amino acids are linked together via a peptide bond to form larger molecules. In this process, the carboxy group of one amino acid reacts with the amino group of another amino acid in a condensation reaction. However, an aqueous solution is unfavorable for the formation of amide bonds [34] and the reaction can, therefore, not be performed under normal conditions without catalysis. An alternative to the formation of peptides by stringing together individual amino acids via peptide bonds could be the formation of amino acids using simple molecules directly from the previous amino acid. Amides could play a central role in this scenario, where a functional peptide can be formed without being dependent on the unfavorable condensation reaction. In the context of the origin of life, the formation of amides has been shown in wet–dry cycles [35,36] or in reactions coupled to pyrite formation [37].

In previous works, we demonstrated the formation of fatty acids [38,39] and intermediates of existing carbon fixation cycles [40] using acetylene and carbon monoxide as carbon sources under simulated volcanic hydrothermal conditions. Nitrogen was successfully introduced into the system via ammonia, as shown by the formation of pyrrole, another essential unit in the biochemistry of modern life [41]. In these reactions, transition metal sulfides served as catalysts, in accordance with the iron–sulfur world theory of Günter Wächtershäuser [19]. In contrast to the typical cyanide-based Strecker scheme, we now show the simultaneous metal-catalyzed formation of amino acids from acetylene, carbon monoxide and ammonia under simulated hydrothermal conditions, again demonstrating the synthetic importance of acetylene for prebiotic chemistry. Moreover, we detected various amides under these aqueous conditions underlining the putative role of amide functionalities in the evolution of peptides and proteins.

## 2. Materials and Methods

All chemicals were purchased from Sigma Aldrich GmbH (Steinheim, Germany) in the highest purity available. Acetylene 2.6 (acetone free) was purchased from Linde AG (Pullach, Germany), and CO 2.5 and argon 4.6 were purchased from Westfalen AG (Münster, Germany).

Experiments were performed as published previously [41]. Briefly, in a typical run (run 1, Table S2), a 125 mL glass serum bottle was charged with 1.0 mmol NiSO_4_ · 6 H_2_O and 1.0 mmol NH_4_Cl and closed with a gas tight silicon stopper.

The bottle was evacuated three times and filled with argon, finally resulting in a de-aerated state. Subsequently, the bottle was filled with argon-saturated water, 1 M Na_2_S solution and 1 M NaOH solution, resulting in a total reaction volume of 5 mL. In this mixture, a precipitate of black NiS was immediately formed due to its low solubility constant of 1 ×10^−22^ [42,43] in aqueous solution. Finally, 60 mL of acetylene gas and 60 mL of CO were added. Reactions were carried out at 105 °C using reaction times up to seven days. Variations were achieved through the addition of different volumes of NaOH or Na_2_S solution and the use of FeSO_4_ or CoSO_4_ instead of or additionally to NiSO_4_. After the defined reaction time, 1 mL of the reaction mixture was freeze dried and derivatized with 0.5 mL acetonitrile and 0.5 mL *N*-*tert*-butyldimethylsilyl-*N*-methyltrifluoracetamide (MTBSTFA) at 70 °C for one hour.

Stable isotope precursors (^13^CO, ^13^C_2_-Acetylene and ^15^ NH_4_Cl) were used to elucidate the composition of the products. ^13^CO and ^15^ NH_4_Cl were directly added instead of their analogs. ^13^C_2_-Acetylene gas was obtained by adding tetra-*n*-butylammonium fluoride (TBAF) to solid ^13^C_2_-(trimethylsilyl)acetylene in an evacuated serum bottle via a syringe. The resulting ^13^C_2_-acetylene gas was then used for experiments.

The TBDMS derivatives of the products were analyzed by GC-MS using a GC-2010, coupled with MS-QP2010 Ultra (Shimadzu GmbH, Duisburg, Germany) with a 30 m × 0.25 mm × 0.25 µm fused silica capillary column (Equity TM5, Supelco, Bellefonte, PA, USA) and an AOC-20i auto injector.

The applied temperature of the column oven was as follows: 0–6 min at 90 °C; 6–25 min at 90–310 °C, 10 °C/min; injector and transfer temperature were kept at 260 °C.

Identification was performed by comparison of retention times and mass spectra of purchased reference compounds, as well as with data from the National Institute of Standards and Technology (NIST) spectral library. Retention times are given in Table S1.

Quantification was performed by external calibration using a solution of alanine with different concentrations.

For comparison, blank runs with argon instead of acetylene and runs without a transition metal compound were performed.

## 3. Results

We reacted acetylene, carbon monoxide and ammonia under demanding anaerobic, aqueous conditions at 105 °C for up to 7 days. As transition metal catalysts, FeS, NiS, CoS and mixtures of them were used, which were freshly prepared in situ from metal sulfates and sodium sulfide.

Under similar conditions, mainly unsaturated, odd numbered carboxylic acids from formic acid up to nonadecenoic acids were detected [38,39]. We now show the formation of carboxylic acid amides up to a chain length of C_5_ and the simultaneous formation of amino acids (Table 1). Runs with ^13^CO, H^13^C≡^13^CH and ^15^NH_4_Cl obtained these products as genuine reaction products and it could be seen that they were composed from the starting materials. In the absence of NiS, FeS or CoS, these amides and amino acids were not formed.

Specifically, we detected α-alanine, β-alanine, glycine, aspartic acid and β-homoserine and 13 amides including formamide, propionamide and succinamic acid (Table 1). All substances were analyzed as their corresponding *tert*-butyldimethylsilyl (TBDMS) derivatives by GC-MS.

Further condensation to detectable amounts of peptides under otherwise similar conditions would probably require higher concentrations [44,45]. In an origin of life scenario, this could be achieved through surface bonding on the catalyzing mineral [46], thermal concentration in hydrothermal rock pores [47] and dehydration–hydration cycles [48]. Nevertheless, the detection of surrogate amides provides evidence for the one-pot formation of amides and amino acids from acetylene, CO and ammonia in a hydrothermal environment. It is tempting to speculate that this scenario could support further evolution into peptides on the early Earth, avoiding the unfavorable condensation of amino acids.

**Figure 1:**
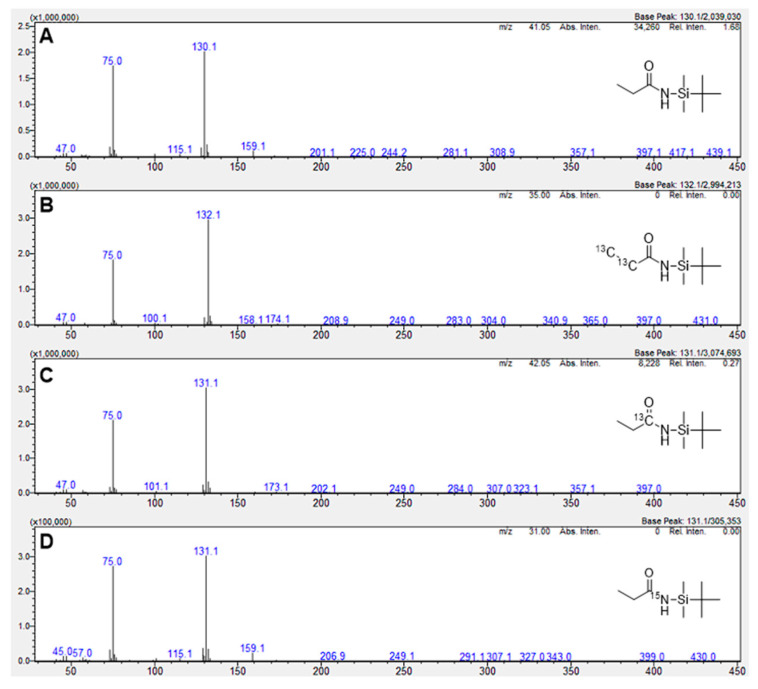
Different mass spectra of propionamide. (**A**) is from run 1 (Table S2), (**B**) is from a run with ^13^C_2_H_2_, (**C**) is from a run with ^13^CO and (**D**) is from a run with ^15^NH_4_Cl. The typical mass of *m*/*z* = 130 results from the loss of the t-butyl group from TBDMS.

**Figure 2:**
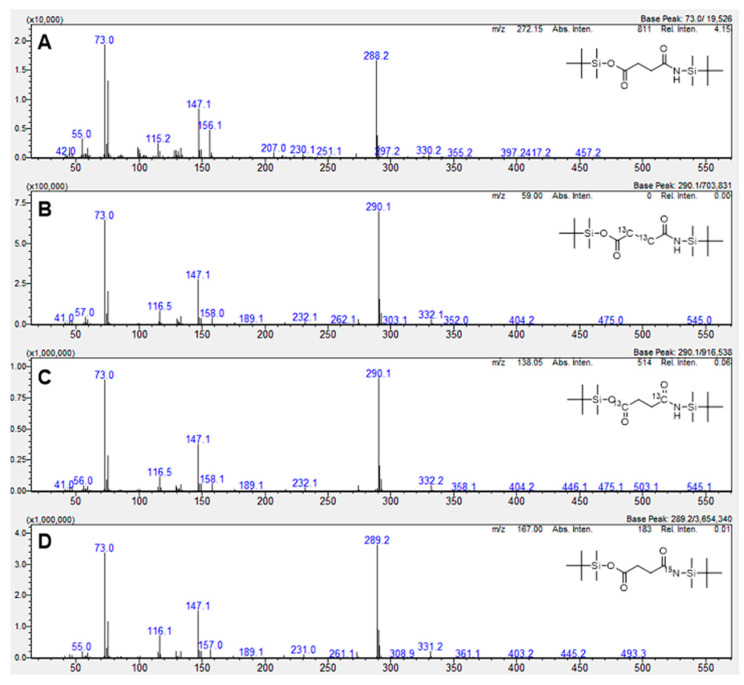
Different mass spectra of succinamic acid. (**A**) is from run 1 (Table S2), (**B**) is from a run with ^13^C_2_H_2_, (**C**) is from a run with ^13^CO and (**D**) is from a run with ^15^NH_4_Cl. The typical mass of *m*/*z* = 288 results from the loss of the t-butyl group from the TBDMS.

**Figure 3:**
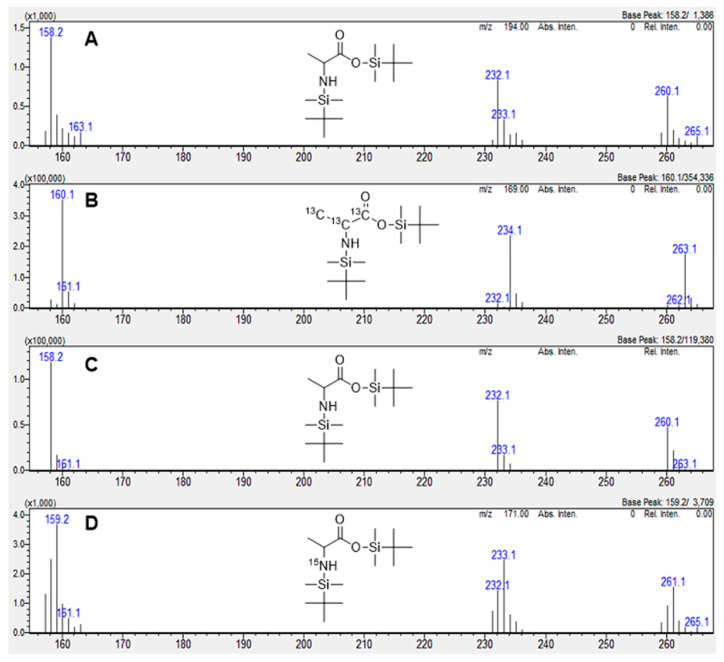
Different mass spectra of alanine. Measurement was performed in single ion monitoring (SIM) mode. Shown are the masses of typical fragments of TBDMS-derivatized amino acids. In the case of alanine these are *m*/*z* = 260, *m*/*z* = 232 and *m*/*z* = 158. (**A**) is from run 1 (Table S2), (**B**) is from a run with ^13^C_2_H_2_, (**C**) is from a run with ^13^CO and (**D**) is from a run with ^15^NH_4_Cl.

**Figure 4:**
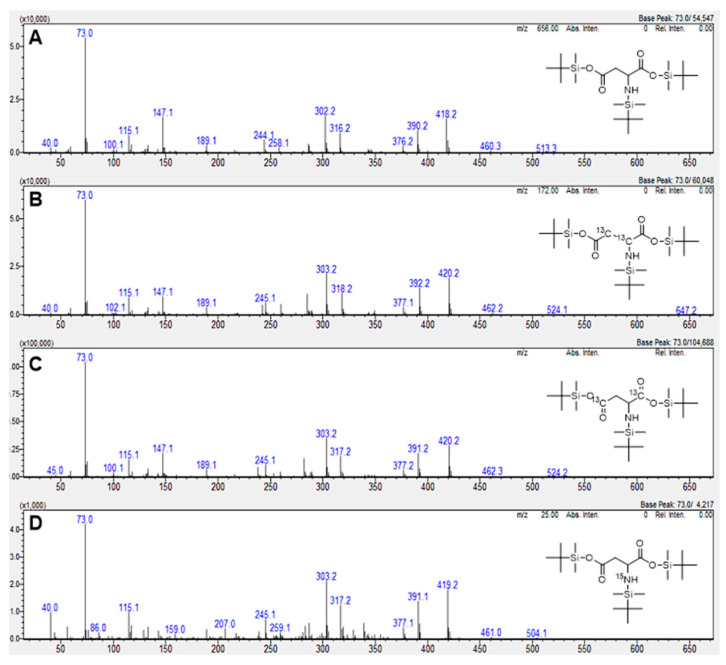
Different mass spectra of aspartic acid. (**A**) is from run 1 (Table S2), (**B**) is from a run with ^13^C_2_H_2_, (**C**) is from a run with ^13^CO and (**D**) is from a run with ^15^NH_4_Cl. Typical mass fragments of TBDMS-derivatized aspartic acid are *m*/*z* = 418, *m*/*z* = 390 and *m*/*z* = 302.

For more a more specific study, two amino acids and two amides were chosen as representatives. In Figure 1, Figure 2, Figure 3 and Figure 4 the mass spectra of differently labelled propionamide (Figure 1), succinamic acid (Figure 2), alanine (Figure 3) and aspartic acid (Figure 4) are shown. In each Figure A shows the corresponding spectra of the unlabeled compounds; B shows the spectra of the reaction products in which ^13^C-acetylene was used; C, in which ^13^CO was used, and D, in which ^15^N-labelled ammonia was used. Propionamide, succinamic acid and aspartic acid, which were used in high amounts, were measured in SCAN mode. Due to the comparatively low yield of alanine, we used single-ion monitoring (SIM) to show the labelling patterns of alanine. Here, only the typical masses of TBDMS- alanine, which are *m*/*z* = 260, *m*/*z* = 232 and *m*/*z* = 158, were measured (Figure S1). The most intensive mass peak usually represents the fragment lacking a t-butyl group (M-57^+^). In the case of propionamide, succinamic acid and aspartic acid these are *m*/*z* = 130, *m*/*z* = 288 and *m*/*z* = 418, respectively. It should be noted that peptides or peptide-like assemblies could not be observed.

**Scheme 2:**
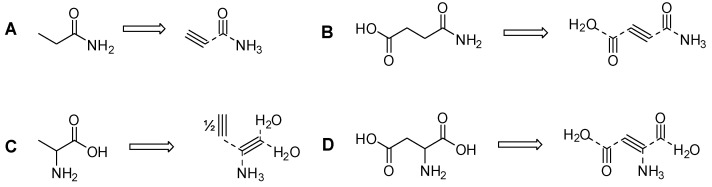
Retrosynthesis of propionamide from one molecule of ammonia, acetylene and carbon monoxide (**A**); succinamic acid from one molecule of ammonia, one molecule of acetylene, two molecules of carbon monoxide and one molecule of water (**B**); alanine from one molecule of ammonia, one and a half molecules of acetylene and two molecules of water (**C**); and aspartic acid from one molecule of ammonia and acetylene and two molecules of carbon monoxide and water (**D**).

Based on the specific mass data for the reaction products obtained from the experiments with different stable isotope labelled precursors, the starting materials from which the individual compounds were synthesized could be identified. It turned out that propionamide was formed from one molecule of acetylene, carbon monoxide and ammonia, succinamic acid from one molecule of acetylene, two molecules of CO and one molecule of ammonia, alanine from one and a half molecule of acetylene, two molecules of water and one molecule of ammonia, and aspartic acid from one molecule of acetylene, two molecules of carbon monoxide, two molecules of water and one molecule of ammonia (Scheme 2). Interestingly, all of the carbon atoms in alanine came from acetylene. In contrast, β-alanine was formed from one molecule of acetylene and one molecule of carbon monoxide (Table 1).

Different reaction parameters were investigated. Tables S2–S5 show all of the reactions which were performed to elucidate the influence of the metal ion catalysts, pH value, reaction time and the amount of Na_2_S in the reaction mixture.

**Figure 5:**
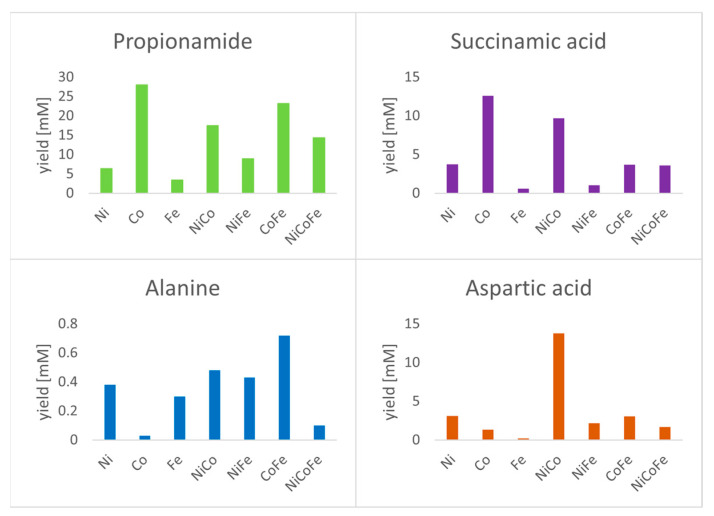
Formation of propionamide, succinamic acid, alanine and aspartic acid with different metal ions as metal sulfide catalysts. Mixtures of transition metal catalysts contain 50/50 (mol%) or 33/33/33 (mol%) of the corresponding metal-sulfides, respectively.

As shown in previous works [41], only metal sulfides, which were freshly formed in situ from metal sulfates and sodium sulfide, were catalytically active in our reaction setup. To analyze the role of the metal sulfide catalysts, we used NiSO_4_, CoSO_4_ and FeSO_4_ as well as 50/50 (mol%) mixtures of two of them and a 33/33/33 (mol%) mixture of all three transition metals to form the respective sulfides. In previous works [41], nickel sulfide showed the best yields in product formation. However, in the case of propionamide and succinamic acid, cobalt sulfide or mixtures of the sulfides gave the best yields. In the case of alanine and aspartic acid, nickel sulfide or sulfide mixtures were superior catalysts (Figure 5).

**Figure 6:**
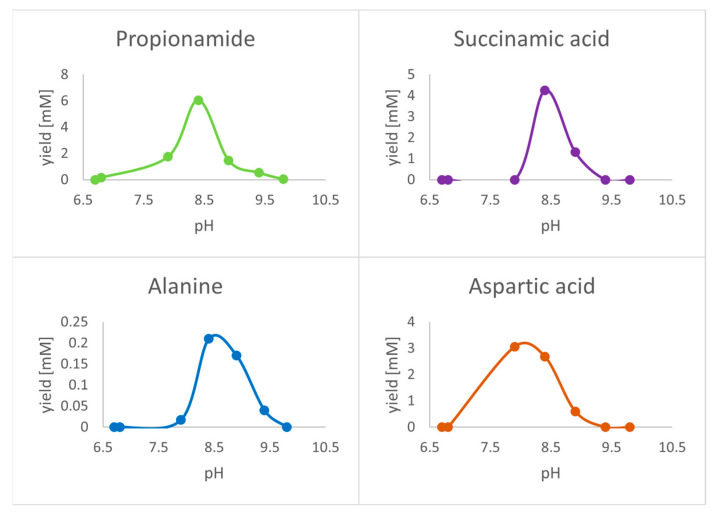
Formation of ***propionamide (green), succinamic acid (violet), alanine (blue) and aspartic acid (orange)*** in the presence of NiS at different pH values. pH values were measured at the end of the reaction time.

Different pH values were achieved by adding NaOH to the reaction mixtures (Table S5). Propionamide, succinamic acid, aspartic acid and alanine showed maximum yields at about pH 8.4 (Figure 6). At acidic pH values, no formation of amides and amino acids could be detected. At pH values > 9.0 the formation of amides and amino acids both rapidly decreased.

**Figure 7:**
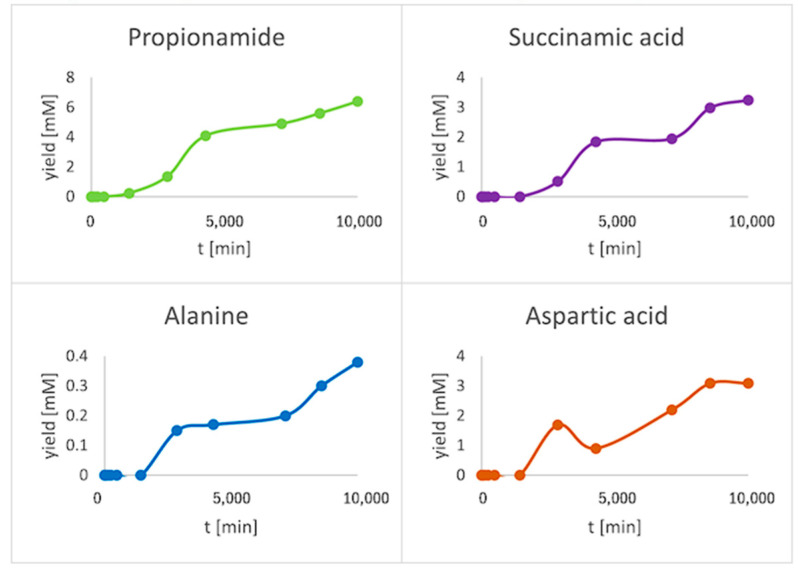
Time-dependent NiS catalyzed formation of ***propionamide (green), succinamic acid (violet), alanine (blue) and aspartic acid (orange)*** from acetylene, carbon monoxide and ammonia.

The formation of amides and amino acids was detected in reaction times ranging from 0 min to 7 days (Figure 7). However, product formation started comparatively slowly and constantly increased until the reaction was stopped at 7 days. According to our data, it seems promising to increase the reaction time beyond 7 days. However, all of the gases in the serum bottle were consumed after this time. Filling up the missing gas volume with CO and acetylene after that point showed no increase in product formation. This can probably be explained by a deactivation of the catalyst during the reaction period. 

**Figure 8:**
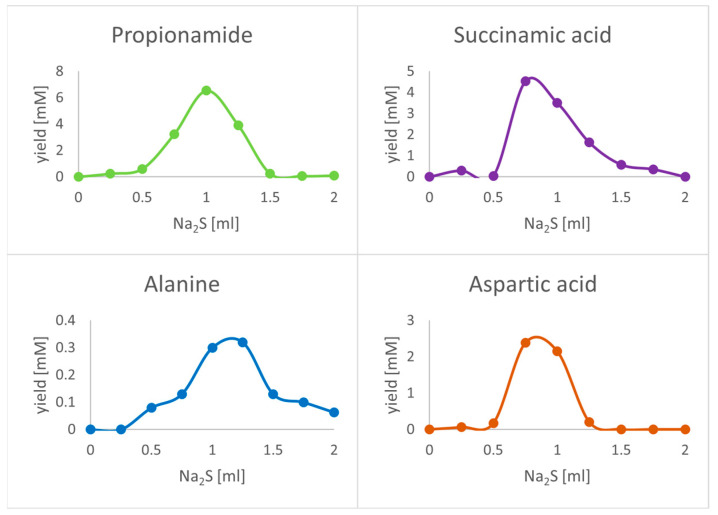
Formation of ***propionamide (green), succinamic acid (violet), alanine (blue) and aspartic acid (orange)*** with different concentration of Na_2_S and therefore different concentration of active catalysts.

As was shown before [41], metal sulfate alone was not catalytically active in our setup. Therefore, the concentration of catalyst in the reaction can be controlled by changing the amount of Na_2_S. As expected, an equimolar concentration of metal sulfate and sodium sulfide showed the best yields of amino acids and amides (Figure 8). Lesser concentrations of sulfide resulted in lesser amounts of active catalysts. Higher concentrations also had a negative effect, which was probably due to their effect on the pH value.

## 4. Discussion

The synthesis of peptide bonds in non-biological systems is, despite recent success [49,50], a challenging issue [51]. These problems are even more serious considering that, in an origin of life scenario, complex protecting groups and appropriate solvents are not available.

Supported by stable isotope labelling, here, we provided proof of the formation of simple amides, potential synthons for more complex peptides, in abiotic reactions starting from acetylene, ammonium chloride, carbon monoxide and metal sulfides under aqueous conditions at 105 °C. The best yields were achieved at a pH of 8.4, equimolar concentrations of metal sulfates and sodium sulfide and seven days of reaction time. Different metal sulfides and mixtures thereof showed catalytic potential in the formation of amino acids and simple amides. The conditions of our experiments were chosen to fit an Hadean scenario, where life possibly emerged near volcanic exhalations [52] or hydrothermal vents [53].

**Scheme 3:**
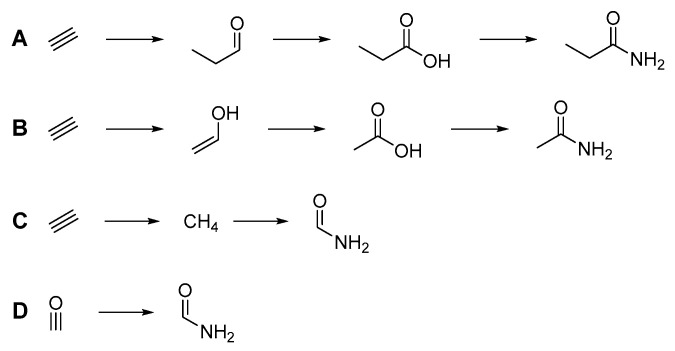
Formation of amides from acetylene as starting material. Shown is the formation of propionamide (**A**), acetamide (**B**) and formamide (**C**). (**D**) shows the simultaneously occurring reaction of CO into formamide.

Based on the ^13^C label distribution in the detected products, three mechanisms of amide formation from acetylene, carbon monoxide and ammonia can be postulated (Scheme 3). The dominant mechanism is the carbonylation or double carbonylation of acetylene, followed by amide formation leading to, e.g., propionamide and succinamic acid (Scheme 3A). A second pathway includes the hydration of acetylene, followed by oxidation and amination, leading to, e.g., acetic acid amide (Scheme 3B). In a third reaction pathway, the cleavage of acetylene is involved, as reflected by the about 66% ^13^C enrichment in formic acid amide when ^13^C labeled acetylene was used as starting material (Scheme 3C). The residual 33% represents a formic acid amide derived from CO (Scheme 3D). We hypothesize that in our experiments, all of the reaction steps took place in the reaction sphere of the metal sulfide precipitates.

**Scheme 4:**
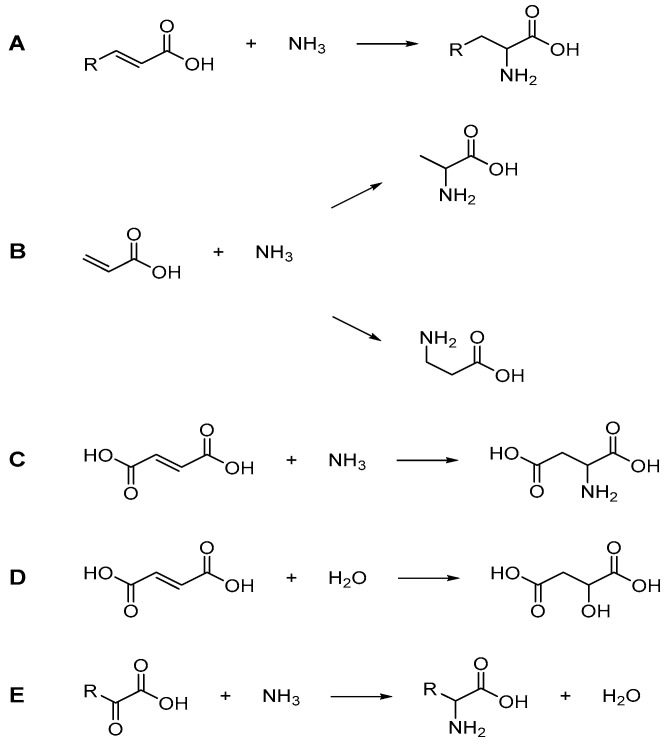
Possible mechanisms of the formation of amino acids in our setup. (**A**): proposed mechanism of NH_3_ addition to α, β unsaturated carboxylic acids. (**B**,**C**): mechanism for the example of α, β alanine and aspartic acid. (**D**): competing reaction with the addition of water and (**E**): another possible mechanism via reductive amination of α-ketoacids.

Amino acids can be formed from α, β unsaturated carboxylic acids through the addition of NH_3_ (Scheme 4A), leading to α and β alanine (Scheme 4B) and aspartic acid (Scheme 4C), a reaction which has to compete with the addition of H_2_O (Scheme 4D) or the reductive amination of α-ketoacids (Scheme 4E) [54].

As mentioned before, the difference in the synthesis of alanine and β-alanine is particularly significant. The carbon atoms of β-alanine originate from one molecule of acetylene and one molecule of carbon monoxide. In contrast, all of the carbon atoms in α-alanine are from acetylene. Interestingly, evolution did not choose the more available anti-*Markownikow* product, β-alanine, as one of its basic building blocks, but rather preferred an alternative route to the *Markownikow* product, α-alanine. This could indicate an early form of control over life processes through the preference for a less present reaction product.

We would like to mention that our analytical setup did not discern between the D and L forms of amino acids. We assume, in the first instance, the formation of racemic mixtures which, in consecutive steps, are selected by different binding constants on the catalytic surfaces [19]. The formation of homochiral peptides and their stability in the Earth’s crust was recently discussed by S. Toxvaerd [55].

The carbon sources in our reaction network were acetylene and carbon monoxide. Acetylene would have been commonly available in an early world scenario as it can be formed by volcanic processes [56] and/or from CaC_2_ in a reaction with water [57]. Acetylene is also considered an important reagent for the formation of smaller molecules containing carbon atoms in interstellar chemistry [58,59]. Acetylene can form smaller hydrocarbons, polyenes and benzenes and therefore lead to a variety of possible carbon precursors for early biochemistry [60]. In our experiments the labelling patterns of the products show the utilization of acetylene as the main carbon source.

Here, we show that all the transition metals, nickel, cobalt and iron, and even mixtures, can serve as potential catalysts in prebiotic reactions. All three can catalyze different reactions to different degrees, which is an indication that different biochemical compounds may have developed from them. These are used for different reactions in modern biochemistry. The natural availability of these metals on early Earth supports this hypothesis. Iron, nickel and cobalt are commonly found in the crust of the Earth [61,62].

Iron, as the most abundant mineral out of these three transition metals, was shown to have the best catalytic properties in earlier studies on reductive amination [54] and CO_2_ reduction [63]. In this study, we report enhanced results for amide and amino acid formation by using nickel, cobalt and mixtures containing cobalt as one constituent. To date, only a limited number of studies have dealt with the electrochemical and catalytic properties of transition metals in an origin of life context and predictions are hard to give. A summary of recent studies is given by de Graaf and Li [64,65]. However, the relevance of all three transition metals as catalysts is supported by extant biochemistry. For example, iron in FeS clusters and hemoglobin enable redox reactions and oxygen transport, respectively. Nickel is important for hydrogen activation and in cofactor F430 for Methanogenesis. Cobalt is contained in coenzyme B12, a cofactor in enzymes that are necessary in the metabolism of amino acids.

As mentioned before, the amino acids are most likely synthesized via a Strecker reaction in a prebiotic environment. We show here that the formation of amino acids is also possible using ammonia only without cyanide, which is necessary for the Strecker reaction. Ammonia, however, does not fit in a relatively oxidized atmosphere, which is assumed for early Earth [66,67]. But, ammonia could be formed out of NO_3_^−^ due to reduction driven by FeS/H_2_S [68]. Nitrate, in this scenario, could be formed from atmospheric N_2_ and CO_2_ through electric discharges under oxygen free conditions [69] and subsequently be dissolved in the ancient ocean.

The formation of peptides is an endergonic process, which means that it does not occur spontaneously under aqueous conditions. This reaction occurs with a decrease in entropy and is so energetically unfavorable that the equilibrium constant K_syn_ for the combination of two amino acids is <10^−5^ [34]. Therefore, in modern synthetic pathways, amino acids must first be activated. To make matters worse, in a prebiotic scenario, an aqueous environment is essential, concentrations are low and activating agents are not available. This makes the condensation reaction even more difficult. Here, we can show the formation of amides, carrying the functionality of dipeptides, in water. In an origin of life context, this finding is important, because we now demonstrate that amides/peptides are not necessarily formed by the condensation of a carboxy group with an amine, but could also be synthesized directly from carbon monoxide, acetylene and ammonium. This pathway avoids the adverse circumstances of peptide formation from individual amino acids and underlines the importance of acetylene in origin of life syntheses.

## Figures and Tables

**Table 1:** Amino acids and amides formed from acetylene (2.71 mmol), ammonia (1.00 mmol) and carbon monoxide (2.68 mmol) in the presence of NiS (1.00 mmol) in 5 mL H_2_O at 105 °C after 7 days. All substances were analyzed as their corresponding TBDMS derivatives. Number of carbon and nitrogen atoms derived from the reactants are identified by stable isotope labelling as indicated.

Compound	# C	# N	^13^C_2_H_2_	^15^NH_4_Cl	^13^CO
Amino acids					
Glycine	2	1	2	1	-
Alanine	3	1	3	1	-
β-Alanine	3	1	2	1	1
Aspartic acid	4	1	2	1	2
β-Homoserine	4	1	2	1	2
Amides					
Formamide	1	1	1	1	-
Urea	1	2	-	2	1
Acetamide	2	1	2	1	-
Acrylamide	3	1	2	1	1
Propionamide	3	1	2	1	1
β-Alanine amide	3	1	2	2	1
Succinamic acid	4	1	2	1	2
Fumaramic acid	4	1	2	1	2
Pentenoic amides	5	1	4	1	1
Pentanoic amide	5	1	4	1	1
2-Aminobenzamide	7	2	6	2	1
2,4-Heptadienoic amide	7	1	6	1	1
Benzamide	7	1	6	1	1

## Data Availability

Data is contained within the article or Supplementary Materials.

## Supplementary Materials

The following supporting information can be downloaded at: https://www.mdpi.com/article/10.3390/life14060719/s1, Table S1. Retention time and typical fragment mass of amino acids and amides, Table S2. Propionamide, succinamic acid, alanine and aspartic acid formation based on different metal catalysts, Table S3. Propionamide, succinamic acid, alanine and aspartic acid formation based on different reaction times, Table S4. Propionamide, succinamic acid, alanine and aspartic acid formation based on different amounts of metal sulfide catalysts, Table S5. Propionamide, succinamic acid, alanine and aspartic acid formation based on different pH values, Figure S1. Typical fragments of TBDMS- amino acids in GC/MS experiments using the example of alanine.
