# Supplementary material for: From Zero to Hero: The Cyanide-Free Formation of Amino Acids and Amides from Acetylene, Ammonia and Carbon Monoxide in Aqueous Environments in a Simulated Hadean Scenario
Source: Life (Basel). 2024 Jun 1;14(6):719. doi: 10.3390/life14060719 (PMC11204499; doi:10.3390/life14060719)
Supplement: Supplementary file 1 [file life-14-00719-s001.zip › life-3025506-supplementary.pdf]

Supplemental Materials

Table S 1. Retention times and typical fragment masses of amino acids and amides. 2
Table S 2. Propionamide, succinamic acid, alanine and aspartic acid formation based on different metal catalysts. 3
Table S 3. Propionamide, succinamic acid, alanine and aspartic acid formation based on different reaction times. 3
Table S 4. Propionamide, succinamic acid, alanine and aspartic acid formation based on different amounts of the metal sulfide catalysts 3
Table S 5. Propionamide, succinamic acid, alanine and aspartic acid formation based on different pH values 4

Figure S 1. Typical fragments of TBDMS-amino acids in GC/MS experiments at the example of alanine. 5

Tables

Table S 1. Retention times and typical fragment masses of amino acids and amides. Amino acids and amides were formed from acetylene (2.71 mmol), ammonia (1.00 mmol) and carbon monoxide (2.68 mmol) in the presence of NiS (1.00 mmol) in 5 ml H2O at 105°C after 7 days. Retention times are given for the method described in materials and methods. M-57 is the characteristic mass of each compound after fragmentation of the tert-butyl group from the silylated form.

**Compound**	**Rt [min]**	**[M-57$^{+\cdot}$]**
Amino acids		
Glycine	13.2	246
Alanine	12.9	260
β-Alanine	14.5	260
Aspartic acid	16.7	418
β-Homoserine	19.1	404
Amides		
Formamide	7.1	102
Urea	14.6	231
Acetamide	11.0	116
Acrylamide	8.3	128
Propionamide	8.4	130
β-Alaninamide	14.5	259
Succinamic acid	16.1	288
Fumaramic acid	17.9	286
Pentenoic amides	9.7/10.5/10.6	156
Pentanoic amide	10.6	158
2-Aminobenzamide	10.2	307
2,4-Heptadienoic amide	13.1	182
Benzamide	14.4	178

If not stated otherwise, all runs were performed with 1.00 mmol $NiSO_4$, 1.00 mmol Na_2S, 1 ml NaOH, 60 ml CO (2.68 mmol) and 60 ml acetylene (2.71 mmol) for seven days at 105 °C. A total reaction volume of 5 ml was achieved by adding argon-saturated water. pH

values were measured at the end of the reaction time. Concentrations are given for the total liquid reaction system of 5 ml. Yields are based on the conversion of NH_4Cl.

Table S 2. Propionamide, succinamic acid, alanine and aspartic acid formation based on different metal catalysts. Ni, Co, Fe and mixture of them were used. Metal sulfides were formed *in situ* from metal sulfates and Na_2S. Other parameters as stated above. Defined standard run is marked in red (1mmol NiS catalyst).

	**NiS** [mmol]	**CoS** [mmol]	**FeS** [mmol]	**pH**	**Conc. Prop** [mM]	**Conc. Succ** [mM]	**Conc. Ala** [mM]	**Conc. Asp** [mM]	**Yield Prop** [%]	**Yield Succ** [%]	**Yield Ala** [‰]	**Yield Asp** [%]
**1**	1	-	-	8.8	7.67	0.75	0.05	0.62	3.84	0.37	0.25	0.31
**2**	-	1	-	7.6	36.01	3.27	<0.01	0.86	18.01	1.63	0.02	0.43
**3**	-	-	1	9.2	4.08	0.12	0.04	0.04	2.04	0.06	0.22	0.02
**4**	0.5	0.5	-	8.7	11.72	11.03	0.06	0.43	5.86	5.51	0.29	0.22
**5**	0.5	-	0.5	8.8	27.85	1.19	0.1	0.61	13.92	0.59	0.48	0.31
**6**	-	0.5	0.5	8.4	20.94	10.1	0.06	2.76	10.47	5.05	0.32	1.38
**7**	0.3	0.3	0.3	8.7	17.21	4.07	0.01	0.47	8.6	2.04	0.07	0.23

Table S 3. Propionamide, succinamic acid, alanine and aspartic acid formation based on different reaction times. Other parameters as stated above. Defined standard run is marked in red (7 days, 10080 minutes).

	**time** [min]	**pH**	**Conc. Prop** [mM]	**Conc. Succ** [mM]	**Conc. Ala** [mM]	**Conc. Asp** [mM]	**Yield Prop** [%]	**Yield Succ** [%]	**Yield Ala** [‰]	**Yield Asp** [%]
**8**	0	9.3	<0.01	<0.01	<0.01	<0.01	<0.01	<0.01	0.03	<0.01
**9**	5	9.3	<0.01	<0.01	<0.01	<0.01	<0.01	<0.01	0.01	<0.01
**10**	10	9.3	<0.01	<0.01	<0.01	<0.01	<0.01	<0.01	0.02	<0.01
**11**	30	9.5	<0.01	<0.01	<0.01	<0.01	<0.01	<0.01	0.02	<0.01
**12**	60	9.3	<0.01	<0.01	<0.01	<0.01	<0.01	<0.01	0.03	<0.01
**13**	120	9.3	<0.01	<0.01	0.01	0.02	<0.01	<0.01	0.05	<0.01
**14**	240	9.3	<0.01	<0.01	0.02	<0.01	<0.01	<0.01	0.09	<0.01
**15**	480	9.3	<0.01	<0.01	0.02	<0.01	<0.01	<0.01	0.12	<0.01
**16**	1440	9.2	0.27	0.02	0.03	0.01	0.14	<0.01	0.15	<0.01
**17**	2880	9.1	1.64	0.1	0.03	0.34	0.82	0.05	0.16	0.17
**18**	4320	9	4.91	0.37	0.03	0.18	2.46	0.18	0.17	0.09
**19**	5760	8.6	<0.01	0.39	0.04	0.45	<0.01	0.19	0.2	0.22
**20**	7200	8.6	5.88	0.39	0.04	0.44	2.94	0.2	0.21	0.22
**21**	8640	8.6	6.71	0.6	0.06	0.62	3.36	0.3	0.3	0.31
**22**	10080	8.8	7.4	0.65	0.06	0.62	3.7	0.32	0.31	0.31

Table S 4. Propionamide, succinamic acid, alanine and aspartic acid formation based on different amounts of the metal sulfide catalysts, which were achieved by adding different amounts of Na_2S. Other parameters as stated above. Defined standard run is marked in red (1 mmol Na_sS).

	**NaS** [mmol]	**pH**	**Conc. Prop** [mM]	**Conc. Succ** [mM]	**Conc. Ala** [mM]	**Conc. Asp** [mM]	**Yield Prop** [%]	**Yield Succ** [%]	**Yield Ala** [‰]	**Yield Asp** [%]
**23**	0	6.8	<0.01	<0.01	<0.01	<0.01	<0.01	<0.01	<0.01	<0.01
**24**	0.25	7	0.21	0.06	<0.01	0.01	0.11	0.03	<0.01	<0.01
**25**	0.5	7.1	0.55	<0.01	0.02	0.04	0.27	<0.01	0.08	0.02
**26**	0.75	8	2.92	0.91	0.03	0.48	1.46	0.45	0.14	0.24
**27**	1	8.6	5.88	0.7	0.06	0.62	2.94	0.35	0.31	0.31
**28**	1.25	9.1	3.53	0.21	0.06	0.4	1.76	0.11	0.32	0.2
**29**	1.5	9	0.22	0.12	0.03	<0.01	0.11	0.06	0.13	<0.01
**30**	1.75	9.3	0.05	0.07	0.02	0.04	0.03	0.04	0.11	0.02
**31**	2	9.6	0.08	<0.01	0.01	<0.01	0.04	<0.01	0.06	<0.01

Table S 5. Propionamide, succinamic acid, alanine and aspartic acid formation based on different pH values, which were achieved by adding different volumes of NaOH solution. Other parameters as stated above. Defined standard run is marked in red (1 mL NaOH).

	**NaOH** [ml]	**pH**	**Conc. Prop** [mM]	**Conc. Succ** [mM]	**Conc. Ala** [mM]	**Conc. Asp** [mM]	**Yield Prop** [%]	**Yield Succ** [%]	**Yield Ala** [‰]	**Yield Asp** [%]
**32**	0	6.7	<0.01	<0.01	<0.01	<0.01	<0.01	<0.01	<0.01	<0.01
**33**	0.25	6.8	0.2	<0.01	<0.01	<0.01	0.1	<0.01	<0.01	<0.01
**34**	0.75	7.9	2.11	<0.01	<0.01	<0.01	1.06	<0.01	0.02	<0.01
**35**	1	8.4	7.26	0.85	0.04	0.61	3.63	0.43	0.22	0.31
**36**	1.25	8.9	1.76	0.26	0.03	0.54	0.88	0.13	0.17	0.27
**37**	1.5	9.4	0.66	<0.01	<0.01	0.12	0.33	<0.01	0.05	0.06
**38**	1.75	9.6	0.05	<0.01	<0.01	<0.01	0.02	<0.01	<0.01	<0.01
**39**	2	9.8	<0.01	<0.01	<0.01	<0.01	<0.01	<0.01	<0.01	<0.01

Figures

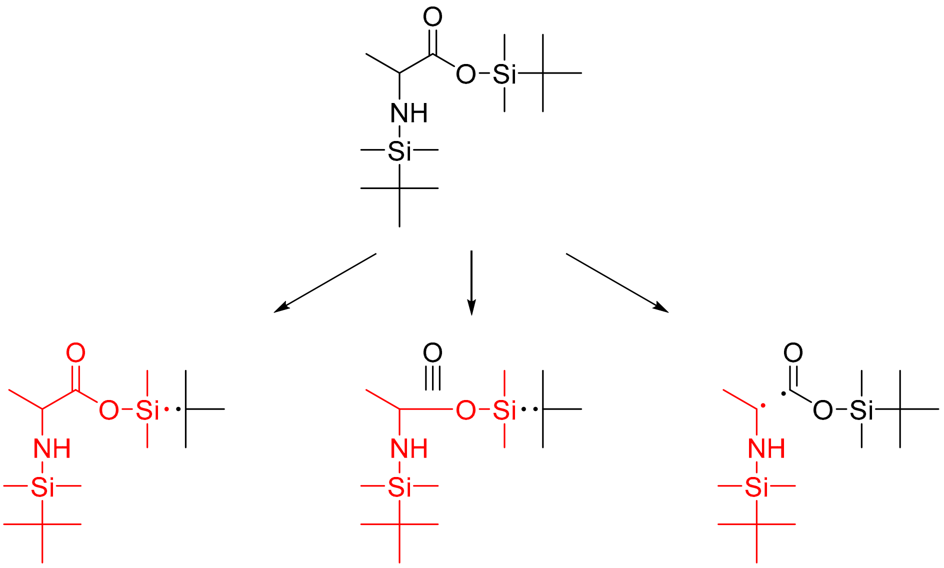

m/z=260 m/z=232 m/z=158

Figure S 1. Typical fragments of TBDMS- amino acids in GC/MS experiments at the example of alanine. Shown in red are the fragments *m/z*=260 ([M-57$^{+\cdot}$], left), *m/z*=232 ([M-85$^{+\cdot}$], middle) and *m/z*=158 ([M-159$^{+\cdot}$], right).
